# Professional identity among forensic medicine students: a cross-sectional study in Jiangsu Province, East China

**DOI:** 10.1186/s12909-025-07387-1

**Published:** 2025-05-27

**Authors:** Kang Wang, Xiaowen Yang, Zixin Han, Kai Li, Youjia Yu, Zhengsheng Mao, Rong Li, Yanfang Yu, Zhiwei Zhang, Yining Huang, Jingjing Ding, Haihong Chen, Feng Chen

**Affiliations:** 1 Department of Forensic Medicine, Nanjing Medical University, Nanjing, 211166 China; 2 Nanjing Medical University Library, Nanjing, 211166 China; 3 School of Health Policy & Management, Nanjing Medical University, Nanjing, 211166 China; 4 College of Journalism and Communication, Central China Normal University, Wuhan, 430079 China; 5 Institution of Medical Education Research, Nanjing Medical University, Nanjing, 211166 Jiangsu China

**Keywords:** Forensic medicine students, Professional identity, Influencing factors, Learning engagement, Academic achievement

## Abstract

**Background:**

Exploring the impact of professional identity on the academic performance of students is crucial for improving teaching effectiveness and educational outcomes in this field. Forensic medicine is a niche interdisciplinary discipline in the medical system. However, current educational literature on professional identity development does not adequately address forensic medicine students.

**Aim:**

This study aimed to assess the professional identity among forensic medicine students, explore factors associated with professional identity, and determine the role of professional identity in shaping students’ learning engagement and their subsequent academic achievements.

**Methods:**

A cross-sectional study was executed for forensic medicine students from a medical university in Jiangsu Province, East China between November and December 2023. Using the method of cluster sampling, 159 undergraduates majoring in forensic medicine were investigated. Data were collected using the demographic questionnaire, and the scales of professional identity, learning engagement, and academic achievement. Linear regression was used to explore professional identity-associated factors. Pearson correlation and mediation analysis were used to analyze the relationship between professional identity, learning engagement, and academic achievement.

**Results:**

The mean score of professional identity was 3.85. Grade (senior: β = 0.353, *P* = 0.004; fifth-year: β = 0.392, *P* = 0.001), student leader experience (β = 0.157, *P* = 0.037), specialty selection (major assignment: β=-0.215, *P* = 0.014), knowledge of the specialty before enrollment (β = 0.095, *P* = 0.033), and current knowledge of the specialty (β = 0.245, *P* = 0.000) were the statistically significant factors influencing professional identity. Professional identity, learning engagement, and academic achievement were positively correlated (*P* < 0.001). Learning engagement played an intermediate role between professional identity and academic achievement, accounting for 49.445% of the total effect.

**Conclusion:**

These findings highlight the pivotal role of professional identity as a strategic mechanism for improving academic achievement in forensic medicine education, with learning engagement serving as the primary mediating factor. As a multifaceted and evolving construct, professional identity is shaped by an interplay of personal, specialty and perception factors. Generating awareness and taking measures among forensic educators to enhance students’ professional identity across stages may be crucial for promoting the quality of forensic talent training.

**Supplementary Information:**

The online version contains supplementary material available at 10.1186/s12909-025-07387-1.

## Highlights

Forensic medicine students exhibited a moderate level of professional identity (Mean Self-Rated Score: 3.85).

Professional identity varies across educational stages, and is affected by various factors such as student leader experience, specialty selection, and knowledge of the specialty.

Professional identity is positively correlated with both learning engagement and academic achievement. Learning engagement plays an intermediate role between professional identity and academic achievement, accounting for 49.445% of the total effect.

**Supplementary Information:**

The online version contains supplementary material available at 10.1186/s12909-025-07387-1.

## Introduction

Professional identity is defined as an individual’s sense of belonging and commitment to a particular professional role [1]. A high professional identity makes college students more enthusiastic and committed to learning, and gives them a stronger professional edge over other students in future career selection [2]. For medical education, professional identity is considered to be as critical as the acquisition of skills and knowledge [3]. It has been further emphasized that the enhancement of virtue-based and behavior-based professionalism in medicine should be achieved through the development of both personal and professional identity formation [4].

Forensic medicine is a specialized medical field that integrates medical techniques with legal and social sciences to investigate crimes. The field of forensic medicine is developing rapidly, gaining wide acceptance in social fairness and justice. Nevertheless, under the influence of traditional concepts (such as taboo around death), lacking understanding about forensic medicine of the public, and religious reasons [5–7], there is public misunderstanding and prejudice for forensic medicine, which leads to a moderately low level of professional identity among forensic medicine students. Research indicates that most students specializing in forensic medicine did not choose it as their first preference in China, with a strong willingness to switch majors [7, 8]. This widespread sentiment has presented challenges to the quality of professional construction and talent training for the forensic disciplines of the State. Meanwhile, university programs play an important role in the formation of professional identity of students, and are essential to understand, shape, and enhance professional identity at the critical period of students’ development [9].

Students’ professional identity was found to be affected by various factors. Gender, education level, grade, family background, practice status, and acceptance of career-planning curriculums are important influencing indicators among nursing students [10, 11]. In addition, professional identity is also a critical factor affecting the learning engagement and academic achievement of students [1, 12]. Uncovering the key factors to improve professional identity, and dissecting the association among professional identity, learning engagement and academic achievement are meaningful for forensic educators, university managers, and government policymakers. These will be helpful for seeking measures to improve the learning quality and effectiveness of forensic medicine students.

As a niche specialty within the medical discipline, forensic medicine was less attended in the concern of professional identity. This lack of academic attention can be particularly challenging for those pursuing careers in specialized fields, where understanding the landscape of professional identity is crucial. Moreover, competence within various forensic cases, including psychology or knowledge of local customs, can impact how students are recognized and valued in the forensic fields [13]. In addition, the role of the professional identity of students will also provide a positive career identity for those pursuing the forensic profession in the future [2], emphasizing the urgency and necessity of enhancing the professional identity of forensic students.

To date, there is a lack of research on the topic of professional identity of forensic medicine students. Accordingly, we aim to assess the current status of professional identity among Chinese forensic medicine students, explore the influencing factors, and dissect the effect of professional identity on learning engagement and academic achievement. These efforts will provide feedback to teachers and university administrators in the planning of continuous professional development support for students, as well as enhancing forensic education qualities.

## Theoretical framework

Based on the causal-chain framework [14], this study analyzed the professional identity of forensic medicine students through the antecedents, mediators and outcomes. The antecedents of professional identity are multi-dimensional. It has been found that professional identity development in medical students at basic science stage was influenced by educational, socioeconomic, personal, and familial ones [15]. Another investigation reported that student leader experience, parents’ education level, different grades, different majors, professional learning conditions, specialty selection, and specialty prospect have an impact on medical students’ professional identity [16]. A review summarized the influencing factors of professional identity among nursing students and nurses, which included personal, family, institutional, and social factors [17].

Professional identity is defined as an individual’s sense of belonging and commitment to a particular professional role [1]; learning engagement describes a learner’s positive and fulfilling mental state focused on the learning process [18]; academic achievement refers to a student’s ability to perform well in academic settings, including their level of success in education [19]. Research shows that professional identity significantly predicts students’ learning engagement [1, 2]. Meanwhile, students who are highly engaged in their learning tend to exhibit a better understanding of course material, improved critical thinking skills, and higher levels of academic achievement [12]. There is also evidence that professional identity exerts influences on the students’ academic achievement, mediated through learning engagement [12].

**Fig. 1:**
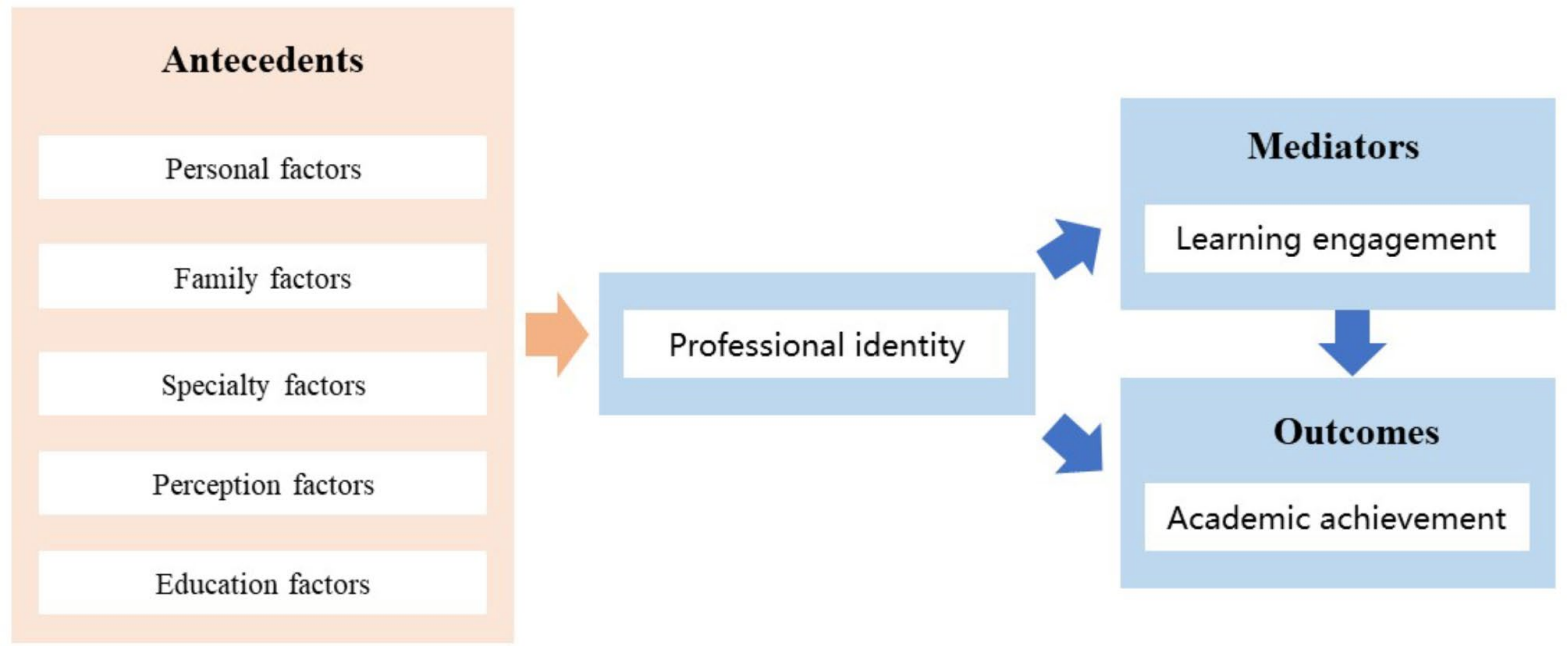
The proposed theoretical framework of this study

The proposed theoretical framework of this study, depicted in Fig. 1, aimed to present personal, family, specialty, perception, and education factors that influence professional identity. It also sought to clarify the potential causal linkages among professional identity, learning engagement, and academic achievement by employing a mediation model.

## Methodology

### Study design and setting

A cross-sectional study was executed from November to December 2023 for forensic medicine students from the Nanjing Medical University, Jiangsu Province, China. Nanjing Medical University is one of the three earliest universities in China to establish the forensic medicine discipline.

### Study population, sampling and data collection

Through cluster sampling, forensic medicine students from all grades were recruited from Department of Forensic Medicine at Nanjing Medical University. The inclusion criteria for participants were: (1) undergraduate students majoring in forensic medicine including freshman, sophomore, junior, senior, and fifth year; (2) who entered university after 2018; (3) voluntary participation in the survey. Finally, 159 forensic medicine students were recruited, which met the inclusion criteria of this study.

Data were collected using offline questionnaires. Some researchers involved in this study were faculty members from the Department of Forensic Medicine, who notified students of the survey schedule and locations. During survey sessions for each grade, researchers explained the study objectives, content, and significance, and addressed students’ questions. Survey sessions were conducted at Nanjing Medical University for first- through fourth-year students, while fifth-year students completed the survey at their internship units in Suzhou, Jiangsu Province, as they were in the internship phase during the study period. After obtaining informed consent, participants completed anonymous questionnaires, which were reviewed immediately upon collection by researchers.

### Instruments

Data were collected using the Demographic and Influencing Factor Questionnaire, the Professional Identity Questionnaire for Undergraduate Students, the Chinese version of the UWES-S (Utrecht Work Engagement Scale-Students), and the Behavioral Performance Scale for Undergraduate Students.

The Demographic and Influencing Factor Questionnaire was developed by the research team based on the literature. Personal factors (gender, age, grade, student leader experience, and only child), family factors (father education level, mother education level, household district, and household economic), specialty factors (college entrance examination performance, first-choice specialty, and specialty selection), perception factors (perception of professional, knowledge of the specialty before enrollment, current knowledge of the specialty, and familiar with forensic practitioners before enrollment) and education factors (learning conditions, professional teaching, moral character education) were included.

A Chinese Version Professional Identity Questionnaire for Undergraduate Students (PIQUS) was developed by Qin [20], containing 23 items spanning four dimensions (cognitive identity, emotional identity, behavior identity, and fitness identity). This scale with good reliability and validity has been widely used in research on the professional identity of medical students in China [12, 16, 21]. All items were scored on a five-point Likert scale, with higher scores indicating a higher level of professional identity.

The Chinese version of the UWES-S was translated by Fang et al. [22]. This 17-item scale contained three dimensions (vigor, dedication, and absorption), with good reliability and validity for assessing Chinese college students [22]. All items were scored on a seven-point Likert scale, with higher scores reflecting greater learning engagement.

A Chinese Version Behavioral Performance Scale for Undergraduate Students was developed by Wang et al. [23]. This 15-item scale contained three dimensions (learning performance, interpersonal facilitation, and learning dedication), with good reliability and validity for assessing Chinese college students [23]. All items were scored on a six-point Likert scale, with higher scores reflecting greater academic achievement.

### Ethical statement

This study was approved by the Ethics Committee of Nanjing Medical University (No: 2023514). Data privacy and anonymity were reassured to the participants.

### Statistical analysis

Frequency and percentage were used to describe the categorical variables. Mean and standard deviation were used to describe the continuous variables. Univariate and multivariate linear regressions were used to determine influencing factors of professional identity. The multivariate linear regression included all significant factors from the univariate linear regression. In the regression models, professional identity was designated as the dependent variable, and personal, family, specialty, perception and education factors as the independent variables. The Kolmogorov–Smirnov test was used to determine the normalcy of dependent variable. Pearson correlation analysis was employed to assess the relationships among professional identity, learning engagement, and academic achievement. Mediation analysis was carried out using Model 4 from PROCESS v4.1. In this analysis, professional identity was designated as the independent variable, learning engagement as the mediator, and academic achievement as the dependent variable.

Data were analyzed with SPSS Statistics. Statistical significance was set as *p* < 0.05.

## Results

### The participant characteristics

Responses were received from 159 forensic medicine students from Nanjing Medical University (Table 1). The analysis involved feedback from participants with an age bracket of 17–24 years, with 34, 33, 33, 29, and 30 students from the freshman, sophomore, junior, senior, and fifth year respectively. The students were predominantly studying at the Public Security Bureau of Jiangsu Province in their fifth professional year. Of the 159 participants, 90 (56.60%) were male, and 69 (43.40%) were female. Nearly 60% of the participants were the only child, due to the one-child policy in China.

**Table 1:** Personal and family situation of forensic medicine students (*N* = 159)

Variables		Frequency	Percentage (%)
**Personal Factors**			
Gender	Male	90	56.60
	Female	69	43.40
Age	17	3	1.89
	18	29	18.24
	19	30	18.87
	20	25	15.72
	21	34	21.38
	22	19	11.95
	23	17	10.69
	24	2	1.26
Grade	Freshman	34	21.38
	Sophomore	33	20.75
	Junior	33	20.75
	Senior	29	18.24
	Fifth Year	30	18.87
Student Leader Experience	No	95	59.75
	Yes	64	40.25
Only Child	No	65	40.88
	Yes	94	59.12
**Family Factors**			
Father Education Level	Primary School and Below	9	5.66
	Junior High School	40	25.16
	High School or Technical Secondary School	50	31.45
	Junior College or Undergraduate	56	35.22
	Postgraduate	4	2.52
Mother Education Level	Primary School and Below	17	10.69
	Junior High School	35	22.01
	High School or Technical Secondary School	56	35.22
	Junior College or Undergraduate	46	28.93
	Postgraduate	5	3.14
Household District	Provincial Capital City	23	14.47
	Prefectural-Level City	52	32.70
	County-Level City	49	30.82
	Township	19	11.95
	Rural Area	16	10.06
Family Economic	Very Bad	9	5.66
	Bad	21	13.21
	Average	103	64.78
	Good	24	15.09
	Very Good	2	1.26

The proportion of junior college/undergraduate was the highest among fathers’ education level (35.22%), and high school/technical secondary school among mothers’ education level (35.22%). Approximately two-thirds of students’ household districts were in a prefecture-level city/county-level city (63.52%), and their family economic status were self-scored average (64.78%).

### Specialty, perception and education situations of forensic medicine students

Over half of the students self-identified as normal performance on the college entrance exam (57.86%, *n* = 92). Although 54.09% of the students have listed forensics medicine as one of their selected specialties, 74.84% of the students did not rank it as their first choice. The professional cognitive assessment showed bipolarization, with 48.43% of the students considering it as a common specialty, while 44.03% considering it as an unpopular specialty. Most students (82.39%) were not familiar with forensic practitioners at the time of choosing a major, which was directly related to their limited knowledge of forensic medicine. The in-depth knowledge or quite knowledgeable about forensic medicine major rate was 37.74% before university enrollment. However, this rate was elevated to 67.92% after varied years spent in university. Furthermore, a majority of students found an agreement support that the learning conditions, professional teaching, and moral character education are satisfied or relatively satisfied, with percentages of 70.44%, 78.61%, and 69.81%, respectively (Table 2).

**Table 2:** Specialty, perception and education situations of forensic medicine students (*N* = 159)

Variables		Frequency	Percentage (%)
**Specialty Factors**			
College Entrance Examination Performance	Lower than normal level	53	33.33
	Normal level	92	57.86
	Higher than normal level	14	8.81
First-Choice Specialty	No	119	74.84
	Yes	40	25.16
Specialty Selection	Autonomous Choice	86	54.09
	Parents’ or Others’ Willing	11	6.92
	Major Assignment	62	38.99
**Perception Factors**			
Perception of Professional	Popular Majors	12	7.55
	Common Majors	77	48.43
	Unpopular Majors	70	44.03
Knowledge of the Specialty before Enrollment	Extremely Unknowledgeable	17	10.69
	Unknowledgeable	36	22.64
	Average	46	28.93
	Knowledgeable	56	35.22
	Extremely Knowledgeable	4	2.52
Current Knowledge of the Specialty	Extremely Unknowledgeable	3	1.89
	Unknowledgeable	10	6.29
	Average	38	23.90
	Knowledgeable	91	57.23
	Extremely Knowledgeable	17	10.69
Familiar with Forensic Practitioners before Enrollment	No	131	82.39
	Yes	28	17.61
**Education Factors**			
Learning Condition	Very Bad	3	1.89
	Bad	4	2.52
	Average	40	25.16
	Good	93	58.49
	Very Good	19	11.95
Professional Teaching	Very Bad	3	1.89
	Bad	1	0.63
	Average	30	18.87
	Good	101	63.52
	Very Good	24	15.09
Moral Character Education (career planning, innovation and entrepreneurship education et al.)	Very Bad	3	1.89
	Bad	6	3.77
	Average	39	24.53
	Good	85	53.46
	Very Good	26	16.35

#### Professional identity, learning engagement and academic achievement of forensic medicine students

**Fig. 2:**
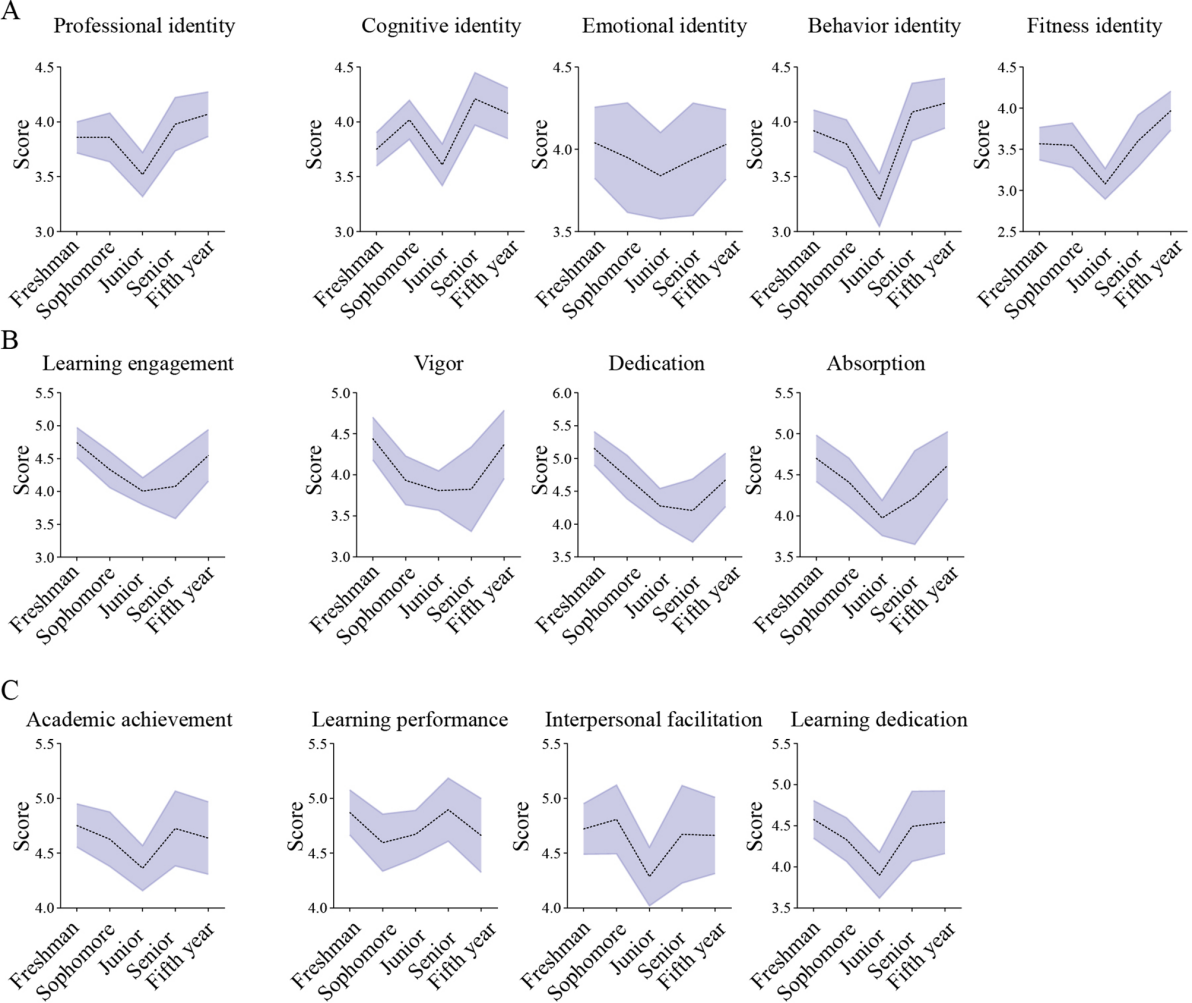
Professional identity, learning engagement and academic achievement of forensic medicine students across different grades. **A** Professional identity of forensic medicine students across different grades. **B** Learning engagement of forensic medicine students across different grades. **C** Academic achievement of forensic medicine students across different grades

Cronbach’s α coefficient of Professional Identity Questionnaire for Undergraduate Students was 0.941. The mean score for professional identity is 3.85. The highest priority was emotional identity (average score of 3.96), followed by cognitive identity (average score of 3.92), behavior identity (average score of 3.84), and fitness identity (average score of 3.55) (Table 3). The current scores of professional identity showed a V-shaped pattern across different grades, reaching the bottom at juniors (Fig. 2A).

**Table 3:** Professional identity, learning engagement and academic achievement of forensic medicine students (*N* = 159)

	Minimum	Maximum	Mean	Standard deviation	Number of items
**Professional identity**	1.48	5.00	3.85	0.59	23
Cognitive identity	1.60	5.00	3.92	0.58	5
Emotional identity	1.13	5.00	3.96	0.76	8
Behavior identity	1.67	5.00	3.84	0.70	6
Fitness identity	1.75	5.00	3.55	0.72	4
**Learning engagement**	1.00	7.00	4.35	0.94	17
Vigor	1.00	7.00	4.08	1.01	6
Dedication	1.00	7.00	4.62	1.03	5
Absorption	1.00	7.00	4.39	1.04	6
**Academic achievement**	2.53	6.00	4.62	0.75	15
Learning performance	2.83	6.00	4.74	0.74	6
Interpersonal facilitation	2.00	6.00	4.63	0.91	6
Learning dedication	1.33	6.00	4.36	0.91	3

The Cronbach’s α coefficient for the Chinese version of the UWES-S and Behavioral Performance Scale for Undergraduate Students were 0.946 and 0.940 respectively. The mean score for learning engagement was 4.35. In this regard, the highest priority was dedication (average score of 4.62), followed by absorption (average score of 4.39), and vigor (average score of 4.08) (Table 3). Overall, scores across different grades showed a U-shaped distribution (Fig. 2B). Meanwhile, the mean score for academic achievement was 4.62, with the highest priority of learning performance (average score of 4.74), followed by interpersonal facilitation (average score of 4.63), and learning dedication (average score of 4.36) (Table 3). The score patterns varied from priorities, including S-shaped in learning performance, V-shaped in interpersonal facilitation and learning dedication (Fig. 2C).

### Influencing factors of professional identity of forensic medicine students

**Fig. 3:**
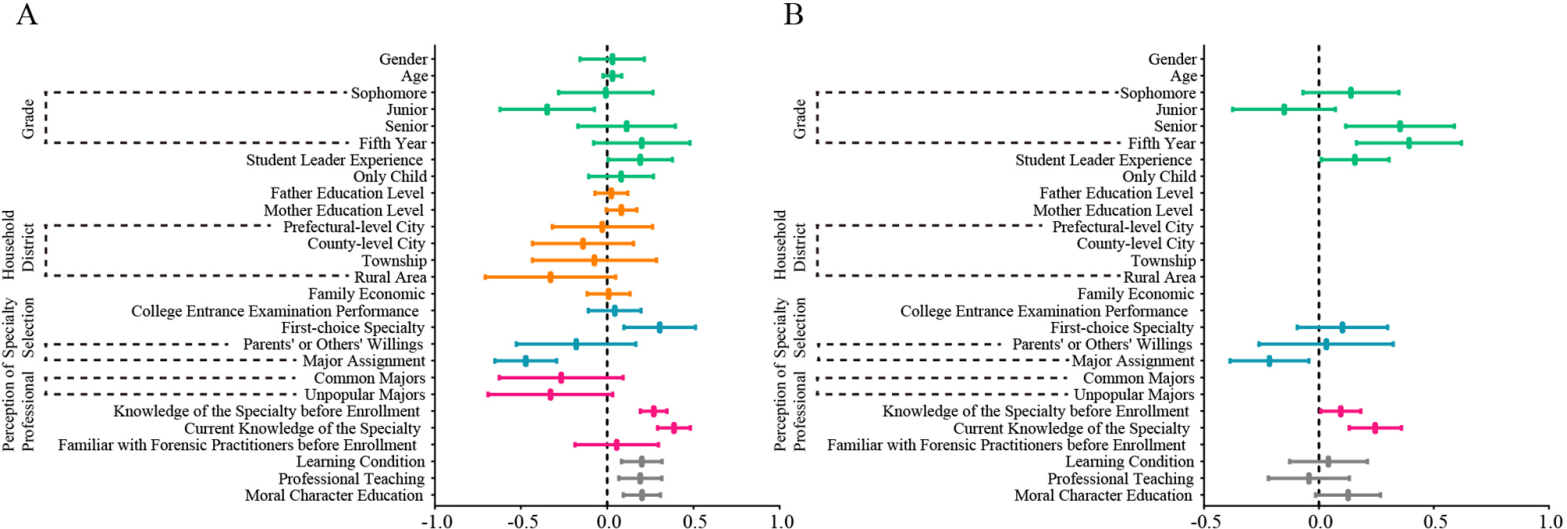
Influencing factors of professional identity of forensic medicine students. **A** Univariate linear regression. **B** Multivariate linear regression. The reference for grade is set as the freshman year, for household district is set as the provincial capital city, for specialty is set as the autonomous choice, for perception of professional is set as the popular majors

To identify influencing factors of professional identity, univariate linear regression and a multivariate linear regression were utilized for analysis. All significant factors from the univariate linear regression were taken into multivariate linear regression for further analysis (Fig. 3). The grade, student leader experience, first-choice specialty, specialty selection, knowledge of the specialty before enrollment, current knowledge of the specialty, learning condition, professional teaching, and moral character education showed statistically significance, these indicators were included in the further multivariate linear analysis (Fig. 3A). The results showed that grade (senior: β = 0.353, *P* = 0.004; fifth year: β = 0.392, *P* = 0.001), student leader experience (β = 0.157, *P* = 0.037), specialty selection (major assignment: β = -0.215, *P* = 0.014), knowledge of the specialty before university entrance (β = 0.095, *p* = 0.033), and current knowledge of the specialty (β = 0.245, *p* = 0.000) have a significant impact on the professional identity of forensic medicine students (Fig. 3B; Table S1).

### Relationship between professional identity, learning engagement, and academic achievement

**Fig. 4:**
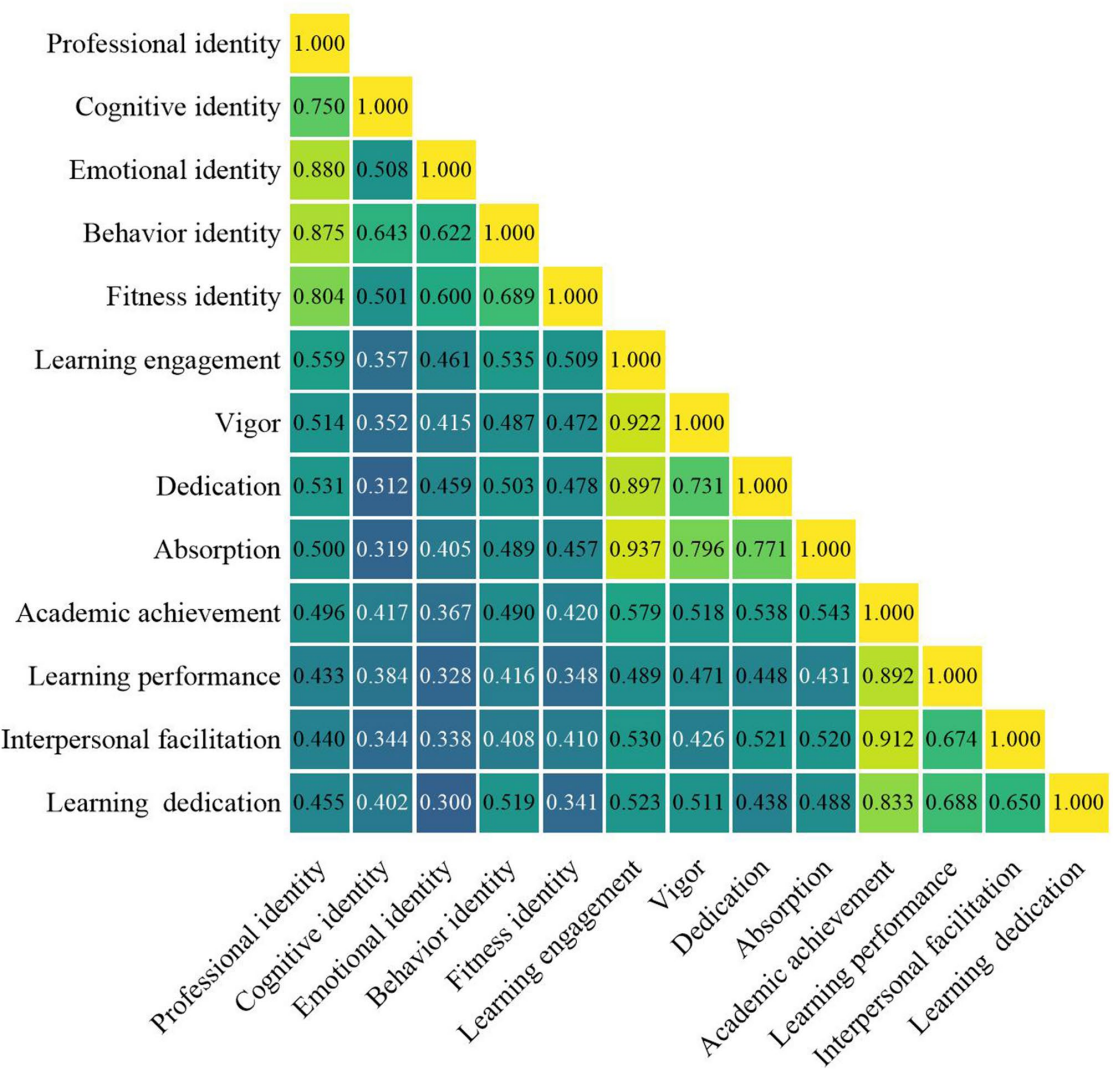
Correlation analysis among professional identity, learning engagement, and academic achievement

The three variables—professional identity, learning engagement, and academic achievement—showed significant positive pairwise correlations (all *P* < 0.001) (Fig. 4). This indicated that a stronger professional identity in forensic medicine students was associated with higher learning engagement or greater academic achievement.

**Fig. 5:**
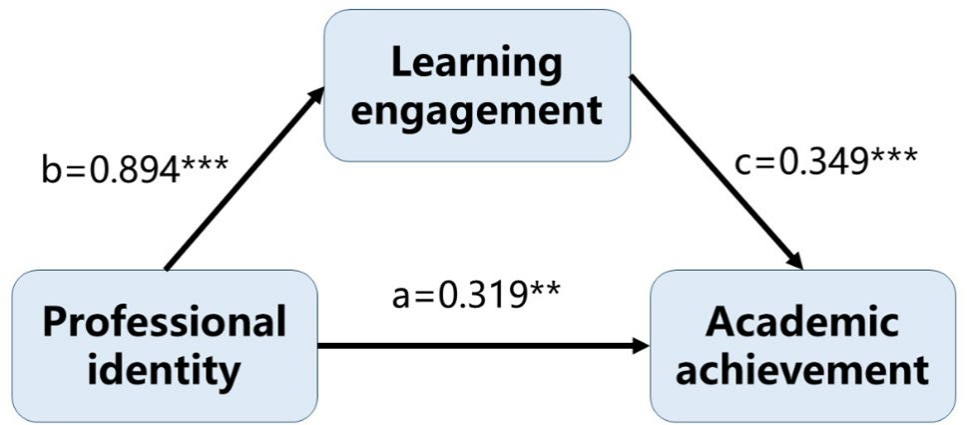
Mediating effect of learning engagement among professional identity and academic achievement in forensic medicine students (*n* = 159). a: the direct effect of professional identity and learning engagement; b: the unstandardized regression coefficient between professional identity and learning engagement; c: the unstandardized regression coefficient between learning engagement and academic achievement. **P < 0.01; ***P < 0.001

The direct effect of professional identity on academic achievement was 0.319 (95% CI [0.128, 0.510]), while the indirect effect was 0.312 (95% CI [0.153, 0.486]) (Table 4; Fig. 5). The mediating effect of learning engagement was significant, and it mediated a partial forward mediating effect. This indicated that professional identity can significantly enhance students’ academic achievement, in which learning engagement played a significant facilitating role in this process.

**Table 4:** Analysis of the mediating effect of learning engagement between professional identity and academic achievement

	Effect	SE	95%CI		Percentage Of Total Effect(%)
			LLCL	ULCL	
Total Effect	0.631	0.088	0.457	0.804	
Direct Effect	0.319	0.097	0.128	0.510	50.555
Indirect Effect	0.312	0.084	0.153	0.486	49.445

SE: Standard Error; LLCL = lower limit of confidence interval; ULCL = upper limit of confidence interval

## Discussion

An individual’s professional identity is the result of a complex psychological process that involves cognitive, emotional, and behavioral components [24]. In this study, the average score for professional identity among forensic medicine undergraduates was 3.85. Another survey focused on the students of the Hangzhou Normal University Division of Health Sciences, and evaluated professional identity for clinical medicine (4.01), stomatology (4.04), nursing (3.81), preventive medicine (3.78), pharmacy (3.55), and health management (3.75) [16]. Compared to these data, the professional identity of forensic medicine undergraduates is lower than that of clinical medicine and stomatology. Forensic professional identity can be further subclassified and ranked into emotional identity, cognitive identity, behavior identity, and fitness identity [20]. This indicates that forensic medicine undergraduates have a stronger emotional identification with the field of study, but perceive a lower alignment between themselves and the profession. Lower self-confidence is the main characteristic of insufficient fitness identity; meanwhile, behavioral identity requires gradual development and enhancement during learning and practice [25]. Therefore, it is essential to put effort into professional education, and cultivate students’ professional skills, which will further enhance professional competence and confidence, and promote a higher professional identity [26].

Grade is an important factor influencing students’ professional identity. The V-shaped pattern across different grades, reaches the bottom at juniors (Fig. 2A), regardless of the total evaluation score for professional identity or the individual dimension scores. An investigation in students from Peking University, Tsinghua University, and Renmin University of China found that students in the middle years of university tend to experience higher levels of depression and stress compared to other grades [27]. Freshman novelty has worn off. After completing their freshman year, many students begin to demonstrate academic competence and setting their individual pathway towards achieving personal and professional goals [28]. An Iranian-based investigation focusing on first-year medical students demonstrated that while first-year medical students have begun embracing their professional roles, their conceptualization of professional identity remains limited in scope [29]. In the subsequent senior years (fourth and fifth years), the curriculum for senior students includes more forensic medicine professional courses. Specialty practice, university dissertations, and other didactic programs allow students to engage in forensic practice, strengthen their understanding of daily forensic work and research frontiers. Deeper knowledge of the specialty makes lower psychological uncertainty of future careers for students, promoting an increase in professional identity [30]. A study of medical students in Japan demonstrated that professional identity scores increased over time, partially with accommodation of clinical practice, suggesting that it could be used not only across professions but also across countries and cultures [31]. Measures to enhance the professional identity have been taken by educators and university managers, for example, a professionalism curriculum at Rice University [32], learning environment improvement at a large public research university in Canada [33], and a medical student-focused humanistic communication model at Hofstra University [34].

Student leader experience was identified to take an active role in professional identity. Class management, interaction between faculty and students, and organizing class activities have greatly improved individual ability and leadership qualities [35, 36]. These in turn can act directly on career planning development, which then reinforces the professional identity [37]. Another additional factor to consider is the specialty selection. Many students did not regard forensic medicine as their first preference, which is one of the key distinctions between students in forensic medicine and clinical medicine. Those who autonomously chose to major in forensic medicine have a higher level of professional identity than those assigned. A stronger emotional affinity, feeling of suitability, and awareness of responsibility are the driving forces of positive cognition and behavior, and are reflected as the higher professional identity. Compared with the bedside specialty (70.2%) [38], the self-chose proportion of forensic medicine is low. There is still improvement space for professional identity through self-chose proportion in forensic medicine students. Knowledge of the forensic specialty is identified to have a positive correlation with professional identity. Professional identity is built upon the students’ knowledge of their specialty. The more knowledgeable about the major, the better their professional identity [39]. Many of the students have little knowledge about the various majors before enrollment, with a lack of career planning. Inadequate knowledge about the specialty will bring obstacles on professional development, which eventually results in reduced professional identity. Conversely, a higher level of professional identity inspires spontaneous study of expertise for the students, forming positive feedback.

The relationship between professional identity and academic achievement is a critical area of research in educational psychology. Our findings of the relationship (Figs. 4 and 5) underscored the importance of nurturing professional identity as a strategic approach to enhancing academic achievement in forensic education, providing a foundation for targeted interventions that foster personal and academic growth. Professional identity plays a significant role in shaping students’ learning engagement and their subsequent academic outcomes [12]. This is particularly evident in fields such as science and health professions, where identity and engagement are closely linked to persistence and success in these disciplines [40]. Moreover, as the findings mentioned above, the development of a professional identity is influenced by various factors, with constantly developing and changing influencing factors. Understanding these dynamics is essential for fostering an environment that promotes both learning engagement and academic achievement. Taken together, a strong professional identity is vital for enhancing learning engagement and academic achievement. It is important for forensic educational institutions or administrations to create supportive environments where students can develop their professional identities, thereby promoting higher levels of learning engagement and achievements in their academic pursuits.

Given that forensic science is a highly specialized discipline, most Chinese provinces typically have only one university offering such programs. This unique distribution enhances the generalizability of our findings across Jiangsu Province. Furthermore, Jiangsu represents an ideal study region as it ranks as both the second most populous province and the largest regional economy in East China, making it a particularly representative area for extension efforts - at minimum within the East China context. A general rule of thumb suggests that multiple linear regression requires a sample size 5 to 10 times the number of predictors to ensure sufficient statistical power [41, 42]. Our multivariate linear regression model included 13 independent variables (including dummy variables), meaning that the sample size of 159 falls within the recommended range and should provide adequate statistical robustness. Subsequent mediation analysis revealed that professional identity had a significant positive effect on learning engagement (0.894), while learning engagement positively influenced academic achievement (0.349), meets the sample requirements for conducting mediation analysis [43].

## Limitation

Forensic medicine education in China differs from that in most developed countries, such as those in the US and Europe. The students who chose forensic medicine were high school graduates, without any accumulation of medical knowledge. Therefore, the research findings may not be generalizable to regions with distinct education frameworks, only potentially generalizable for regions with similar education systems.

## Conclusion

This study demonstrates the significant impact of professional identity on the academic achievement of forensic medicine students, primarily mediated through learning engagement. Professional identity is positively correlated with both learning engagement and academic achievement. Learning engagement plays an intermediate role between professional identity and academic achievement, accounting for 49.445% of the total effect. Thereby, an integrative approach in the curriculum to support professional identity in the middle years of university is important, interventions of integrating mentorship programs and forensic practice early in medical training may be effectiveness. This study also highlights the importance of a conscious professional identity process for forensic medicine students, revealing the need for further focus from educational, medical, and legal administration to assure longitudinal support for forensic medicine education, to promote the quality of forensic talent training.

## Electronic supplementary material

Below is the link to the electronic supplementary material.

Supplementary Material 1

## Data Availability

Data available on request from the authors.
