# Supplementary material for: Professional identity among forensic medicine students: a cross-sectional study in Jiangsu Province, East China
Source: BMC Med Educ. 2025 May 27;25:786. doi: 10.1186/s12909-025-07387-1 (PMC12117931; doi:10.1186/s12909-025-07387-1)
Supplement: Supplementary file 1 — Supplementary Material 1 [file 12909_2025_7387_MOESM1_ESM.docx]

**Table S1 Influencing factors of professional identity of forensic medicine students (N=159)**

|  |  | **univariate linear regression** | | | | **multivariate linear regression** | | | |
| --- | --- | --- | --- | --- | --- | --- | --- | --- | --- |
|  |  | Coeff- |  | 95%CI | | Coeff- |  | 95%CI | |
|  |  | icient | *P* value | LLCL | ULCL | icient | *P* value | LLCL | ULCL |
| **Personal Facotrs** | Gender | 0.030 | 0.755 | -0.157 | 0.216 |  |  |  |  |
|  | Age | 0.031 | 0.266 | -0.024 | 0.085 |  |  |  |  |
|  | Grade |  |  |  |  |  |  |  |  |
|  | Freshman | reference |  |  |  |  |  |  |  |
|  | Sophomore | -0.008 | 0.954 | -0.281 | 0.265 | 0.140 | 0.187 | -0.069 | 0.348 |
|  | Junior | -0.348 | **0.013** | -0.621 | -0.075 | -0.151 | 0.183 | -0.375 | 0.072 |
|  | Senior | 0.113 | 0.430 | -0.169 | 0.395 | 0.353 | **0.004** | 0.116 | 0.590 |
|  | Fifth Year | 0.201 | 0.158 | -0.079 | 0.480 | 0.392 | **0.001** | 0.164 | 0.620 |
|  | Student Leader Experience | 0.192 | **0.043** | 0.006 | 0.378 | 0.157 | **0.037** | 0.010 | 0.305 |
|  | Only Child | 0.081 | 0.397 | -0.107 | 0.268 |  |  |  |  |
| **Family Factors** | Father Education Level | 0.024 | 0.616 | -0.071 | 0.120 |  |  |  |  |
|  | Mother Education Level | 0.083 | 0.066 | -0.006 | 0.172 |  |  |  |  |
|  | Household District |  |  |  |  |  |  |  |  |
|  | Provincial Capital City | reference |  |  |  |  |  |  |  |
|  | Prefectural-Level City | -0.028 | 0.851 | -0.319 | 0.263 |  |  |  |  |
|  | County-Level City | -0.140 | 0.347 | -0.434 | 0.153 |  |  |  |  |
|  | Township | -0.074 | 0.687 | -0.434 | 0.286 |  |  |  |  |
|  | Rural Area | -0.329 | 0.088 | -0.707 | 0.049 |  |  |  |  |
|  | Family Economic | 0.008 | 0.901 | -0.116 | 0.132 |  |  |  |  |
| **Specialty Factors** | College Entrance Examination Performance | 0.044 | 0.576 | -0.110 | 0.197 |  |  |  |  |
|  | First-Choice Specialty | 0.305 | **0.004** | 0.097 | 0.512 | 0.103 | 0.303 | -0.094 | 0.299 |
|  | Specialty Selection |  |  |  |  |  |  |  |  |
|  | Autonomous Choice | reference |  |  |  |  |  |  |  |
|  | Parents' or Others' Willing | -0.180 | 0.306 | -0.526 | 0.166 | 0.032 | 0.831 | -0.261 | 0.324 |
|  | Major Assignment | -0.472 | **0.000** | -0.652 | -0.292 | -0.215 | **0.014** | -0.386 | -0.044 |
| **Perception Factors** | Perception of Professional |  |  |  |  |  |  |  |  |
|  | Popular Majors | reference |  |  |  |  |  |  |  |
|  | Common Majors | -0.267 | 0.145 | -0.626 | 0.093 |  |  |  |  |
|  | Unpopular Majors | -0.329 | 0.074 | -0.691 | 0.032 |  |  |  |  |
|  | Knowledge of the Specialty Before Enrollment | 0.270 | **0.000** | 0.193 | 0.347 | 0.095 | **0.033** | 0.008 | 0.182 |
|  | Current Knowledge of the Specialty | 0.387 | **0.000** | 0.292 | 0.482 | 0.245 | **0.000** | 0.131 | 0.359 |
|  | Familiar with Forensic Practitioners Before Enrollment | 0.055 | 0.655 | -0.188 | 0.298 |  |  |  |  |
| **Education Factors** | Learning Condition | 0.201 | **0.001** | 0.084 | 0.318 | 0.042 | 0.626 | -0.127 | 0.211 |
|  | Professional Teaching | 0.192 | **0.003** | 0.068 | 0.317 | -0.043 | 0.630 | -0.220 | 0.133 |
|  | Moral Character Education | 0.202 | **0.000** | 0.094 | 0.309 | 0.127 | 0.078 | -0.014 | 0.268 |

LLCL = lower limit of confidence interval; ULCL = upper limit of confidence interval. Dependent variable (professional identity) was normally distributed (*P* value of Kolmogorov–Smirnov test was 0.609).
